# Enhanced SCAP Glycosylation by Inflammation Induces Macrophage Foam Cell Formation

**DOI:** 10.1371/journal.pone.0075650

**Published:** 2013-10-16

**Authors:** Chao Zhou, Han Lei, Yaxi Chen, Qing Liu, Lung-Chih Li, John F. Moorhead, Zac Varghese, Xiong Z. Ruan

**Affiliations:** 1 Centre for Lipid Research, Key Laboratory of Metabolism on Lipid and Glucose, Chongqing Medical University, Chongqing, P.R. China; 2 Department of Cardiology, The First Affiliated Hospital of Chongqing Medical University, Chongqing, P.R. China; 3 Centre for Clinical Research, The First Affiliated Hospital of Chongqing Medical University, Chongqing, P.R. China; 4 John Moorhead Research Laboratory, Centre for Nephrology, University College London (UCL) Medical School, Royal Free Campus, London, United Kingdom; 5 Kaohsiung Chang Gung Memorial Hospital and Chang Gung University College of Medicine, Kaohsiung, Taiwan; The University of New South Wales, Australia

## Abstract

Inflammatory stress promotes foam cell formation by disrupting LDL receptor feedback regulation in macrophages. Sterol Regulatory Element Binding Proteins (SREBPs) Cleavage-Activating Protein (SCAP) glycosylation plays crucial roles in regulating LDL receptor and 3-hydroxy-3-methyl-glutaryl-CoA reductase (HMGCoAR) feedback regulation. The present study was to investigate if inflammatory stress disrupts LDL receptor and HMGCoAR feedback regulation by affecting SCAP glycosylation in THP-1 macrophages. Intracellular cholesterol content was assessed by Oil Red O staining and quantitative assay. The expression of molecules controlling cholesterol homeostasis was examined using real-time quantitative RT-PCR and Western blotting. The translocation of SCAP from the endoplasmic reticulum (ER) to the Golgi was detected by confocal microscopy. We demonstrated that exposure to inflammatory cytokines increased lipid accumulation in THP-1 macrophages, accompanying with an increased SCAP expression even in the presence of a high concentration of LDL. These inflammatory cytokines also prolonged the half-life of SCAP by enhancing glycosylation of SCAP due to the elevated expression of the Golgi mannosidase II. This may enhance translocation and recycling of SCAP between the ER and the Golgi, escorting more SREBP2 from the ER to the Golgi for activation by proteolytic cleavages as evidenced by an increased N-terminal of SREBP2 (active form). As a consequence, the LDL receptor and HMGCoAR expression were up-regulated. Interestingly, these effects could be blocked by inhibitors of Golgi mannosidases. Our results indicated that inflammation increased native LDL uptake and endogenous cholesterol de novo synthesis, thereby causing foam cell formation via increasing transcription and protein glycosylation of SCAP in macrophages. These data imply that inhibitors of Golgi processing enzymes might have a potential vascular-protective role in prevention of atherosclerotic foam cell formation.

## Introduction

Atherosclerosis, a maladaptive chronic inflammatory response in the vessel wall, is the primary cause of coronary artery disease, stroke and peripheral vascular disease and it thus represents the most common cause of morbidity and mortality worldwide [1]. Macrophage foam cell formation with cholesterol overloading is the defining pathological characteristic of atherosclerotic plaques [2]. LDL, the major carrier of plasma cholesterol, enters the vessel wall and macrophages by receptor and non-receptor-mediated mechanisms. Increased serum levels of LDL have been most closely correlated with the incidence of cardiovascular disease [3]. Traditionally, scavenger receptors mediated modified LDL (oxidized or glycosylated) uptake is recognized as the major resource for cholesterol accumulation in monocyte-derived macrophages within atherosclerotic plaques [4]. However, recent evidence has challenged this paradigm by showing that loss of receptor-mediated lipid uptake via scavenger receptor A or CD36 pathways does not ameliorate atherosclerosis in hyperlipidemic mice [5]. Our previous studies also showed that the accelerating effects of inflammatory cytokines on lipid droplets accumulation in various peripheral cells such as human mesangial cells (HMCs), vascular smooth muscle cells (VSMCs) and macrophages [6], [7], [8], were not be inhibited by scavenger receptors blocker, but were blocked by LDL receptor (LDLr) specific antibody (MB47) and heparin, which removes LDL bound to the cell surface [7], [8]. This suggests LDLr pathway involvement in lipid accumulation under inflammatory stress.

LDLr, the primary receptor for binding and internalization of plasma-derived native LDL cholesterol and regulation of plasma LDL concentration, was initially considered unimportant in macrophage cholesterol accumulation and foam cell formation because LDLr gene expression in mammalian cells is normally under tight negative-feedback control via Sterol Regulatory Element Binding Protein (SREBP) [9]. In mammalian cells, two SREBP genes encode three different isoforms of SREBPs, known as SREBP-1a, -1c and -2. While SREBP-1a is a potent activator of all SREBP-responsive genes, SREBP-1c preferentially enhances the transcription of genes involved in fatty acid synthesis. Conversely, SREBP-2 preferentially activates genes of LDLr involved in cholesterol uptake and 3-hydroxy-3-methyl-glutaryl- CoA reductase (HMGCoAR) involved in cholesterol biosynthesis [10].

SREBP Cleavage- Activating Protein (SCAP) is a transmembrane protein that serves as a chaperone protein of SREBP2 and sterol sensor, which plays a central role in the SREBP2 activation. When cells are depleted of cholesterol, SCAP delivers the SREBP2 from the endoplasmic reticulum (ER) to the Golgi where it is cleaved by two membrane-bound proteases (site 1 protease and site 2 protease) [11]. Meanwhile SCAP is glycosylated by the sequential action of Golgi enzymes α-mannosidase I, α-mannosidase II and GlcNAc transferase I [12], [13], [14], before recycling to the ER. The sequential cleavages release the active N-terminal fragment of SREBP2 (N-SREBP2) from the Golgi to the nucleus, binding to the sterol regulatory elements in the HMGCoAR and LDLr promoters and activating these genes transcription. When intracellular cholesterol is overloaded, SCAP-SREBP2 complex is retained in the ER and SREBP2 cannot be processed by the proteases in the Golgi. Thereafter the expression of LDLr and HMGCoAR is down-regulated and both cholesterol uptake and de novo synthesis decline. Yuan et al reported that SCAP glycosylation can be decreased by Golgi mannosidase inhibitors, which led to reduced LDLr and HMGCoAR expression and therefore intracellular cholesterol accumulation in HMCs [15]. It seems that SCAP cycling between the ER and the Golgi regulated by Golgi glycosylation is a key process in the feedback regulation of LDLr and HMGCoAR.

Chronic systemic inflammation is an aggravating factor for lipid-mediated peripheral cell injury [16]. A substantial body of evidence has accumulated linking an increased cardiovascular risk in patients with renal failure and immune dysregulation which facilitates activation of chronic inflammatory response [17], [18]. The atherosclerotic patients with elevated levels of acute-phase reactants (reflecting enhanced hepatic production in response to circulating inflammatory cytokines) have a less favorable clinical course than those with normal levels [19]. However, the mechanisms by which activation of the inflammatory response may contribute to atherosclerosis are not fully understood. In the last decades, growing evidences revealed that inflammatory cytokines (i.e. IL-1 and TNF-α) have a direct effect on glycome changes and the cohorts of glycosidases in the Golgi apparatus are the main determining factors of glycome changes [20], [21], [22].

Macrophage is the most important immune response cell and foam cell progenitor. The current experiments were undertaken to investigate whether inflammation disrupts the SCAP mediated feedback regulation for LDLr and HMGCoAR by enhancing SCAP glycosylation by Golgi enzymes, therefore modifies cholesterol homeostasis and promotes cholesterol accumulation in macrophages.

## Materials and Methods

### Cell Culture

Human monocyte cell line (THP-1) (ATCC, no: TIB-202) was cultured in RPMI 1640 medium containing 10% fetal calf serum, 2 mmol/l glutamine, 100 U/ml penicillin, and 100 µg/ml streptomycin. THP-1 was fully differentiated into macrophages by triggered with 160 nmol/l phorbol, 12-myristate, 13-acetate (PMA) for 72 h, and the differentiated THP-1 macrophages were washed extensively with phosphate-buffered saline (PBS) before use in the experiments. Experiments were performed in serum free experimental medium containing RPMI 1640, 0.2% bovine serum albumin (BSA), 2 mmol/l glutamine, 100 U/ml penicillin, 100 µg/ml streptomycin with the anti-oxidants EDTA and butylated hydroxytoluene (BHT) at final concentrations of 100 µmol/l and 20 µmol/l, respectively (Sigma, St. Louis, MO, USA). All reagents for cell culture were obtained from Hyclone (Beijing, China). BSA, PMA, MTT, kifunensine and swainsonine were obtained from Sigma (St. Louis, MO, USA). Recombinant human IL-6 and TNF-α were obtained from SinoBio (Shanghai, China) and PeproTech Asia (USA), respectively.

### LDL Preparation

Fresh unfrozen plasma (200 ml) donated by healthy volunteer was provided by the UK National Health Service Blood and Transplant (NHSBT) in Colindale, London. The study was approved by the NHSBT's Research and Development (R&D) Committee. Our study protocols adhered to the tenets of the Declaration of Helsinki for experiments involving human samples. LDL was isolated from the plasma by sequential density gradient ultracentrifugation as described in our previous publication [23].

### Cytotoxicity Assay by MTT

To evaluate the effect of inflammatory cytokine and Golgi glycosylation enzyme inhibitors on THP-1 macrophage viability, MTT-test has been applied [24]. THP-1 cells were seeded into 96-well plates (2×10^4^ cells per well) and activated by PMA for 72 h. After incubated in serum-free experimental medium for 24 h, the THP-1 macrophage were treated with serum-free experimental medium or experimental medium with 40 ng/ml IL-6 or 50 ng/ml TNF-α in the absence or presence of different concentration of glycosylation inhibitors for 24 h. The cells were treated with MTT, and absorbance was read at 570 nm in a microplate reader (Awareness Technology Inc, USA). Cell viability is expressed as the percentage of absorbance from treated cells compared to untreated cells.

### Morphological Examination

THP-1 macrophages were incubated in chamber slides in serum-free experimental medium or experimental medium with 40 ng/ml IL-6 or 50 ng/ml TNF-α or 25 µg/ml LDL in the absence or presence of glycosylation inhibitors (2.5 ng/ml kifunensine and 2.5 ng/ml swainsonine). After 24 h incubation, the cells were washed three times in phosphate-buffered saline (PBS), fixed for 30 min with 5% formalin solution in PBS, stained with Oil Red O for 30 min, and counterstained with hematoxylin for another 5 min. Finally, the cells were examined by light microscopy (Olympus, Japan). Semi-quantitative analysis of ORO positive staining was performed by the Image-J software.

### Quantitative Measurement of Intracellular Cholesterol

THP-1 macrophages in 6-well plates were cultured in serum-free experimental medium or experimental medium with 40 ng/ml IL-6 or 50 ng/ml TNF-α or 25 µg/ml LDL in the absence or presence of glycosylation inhibitors (2.5 ng/ml kifunensine and 2.5 ng/ml swainsonine) for 24 h. Cells were then washed twice in PBS, intracellular lipids were extracted in chloroform/methanol (2∶1) mix and dried under vacuum, and the total cholesterol (TC) and free cholesterol (FC) content were measured by an enzymatic assay normalized by total cell proteins determined by the Lowry assay [25]. The concentration of cholesterol ester (CE) was calculated using TC-FC.

### Total RNA isolation and real-time quantitative polymerase chain reaction (PCR)

Total RNA was isolated from cultured THP-1 macrophages using RNAiso kit (Takara, Dalian, China) according to the manufacturer's protocol. Total RNA (1 µg) was used as a template for reverse transcription (RT) using a PrimeScript® RT reagent Kit (Takara, Dalian, China). Real time reverse transcription polymerase chain reaction (RT-PCR) was performed in an ABI 7000 Sequence Detection System using SYBR Green dye according to the manufacturer's protocol (Applied Biosystems). All the PCR primers were designed by Primer Express Software V2.0 (Table 1). After the PCR, a dissociation curve (melting curve) was constructed in the range of 60°C to 95°C. Relative amount of mRNA was calculated using the comparative threshold cycle (Ct) method. The amplification efficiencies of the target and reference were shown to be approximately equal with a slope of log input amount to Ct<0.1. Controls consisting of H_2_O or samples that were not reversely transcribed were negative for target and reference.

**Table 1:** The primers for real-time PCR.

Genes	Primers
SREBP2	5′-CGATGCCCTTCAGGAGCTT-3′-sense
	5′-GCGCCAGGAGAACATGGT-3′-antisense
LDLr	5′-CTGTGGGCTCCATAGGCTATCT-3′-sense
	5′-GCGGTCCAGGGTCATCTTC -3′-antisense
HMGCoAR	5′- TCTGGCAGTCAGTGGGAACTATT-3′ -sense
	5′- CCTCGTCCTTCGATCCAATTT-3′ -antisense
SCAP	5′-ACTGGACTGAAGGCAGGTCAA-3′-sense
	5′-GCCTCTAGTCTAGGTCCAAAGAGTTG-3′-antisense
α-Mannosidase I	5′- TGGTATTG GAAGGAACTGGCC-3′-sense
	5′- GCCAGAATACTGCTGCCTCC-3′-antisense
α-Mannosidase II	5′-AATGGGACACTGAACCCCTTC-3′-sense
	5′-CGTTATGGGAATGAGGCACC-3′-antisense
β-Actin	5′-CCTGGCACCCAGCACAAT-3′-sense
	5′-GCCGATCCACACGGAGTACT-3′-antisense

### Protein isolation and Western blots analysis

Identical amounts of protein from cultured THP-1 macrophages extracts or nuclear extracts were denatured and then subjected to electrophoresis on an 8% SDS polyacrylamide gel in a Bio-Rad mini protein apparatus (Bio-Rad Laboratories, Hemel Hempstead, UK). Electrophoretic transfer to nitrocellulose was accomplished at 85 V, 220 mA for 3 h. The membrane was then blocked with 5% skimmed milk for 1–2 h at room temperature and probed with the following antibodies: rabbit anti-human LDLr monoclonal antibody (Abcam, Cambridge, UK), rabbit anti-human HMGCoAR polyclonal antibody (Abgent, Oxfordshire, UK), goat anti–human SREBP2 polyclonal antibody (Santa Cruz Biotechnology, Wiltshire, UK), mouse anti–human SCAP polyclonal antibody (Santa Cruz Biotechnology), and mouse anti-human β-actin polyclonal antibody (Sigma) in antibody dilution buffer (1% BSA in PBST). A rabbit anti-mouse or goat anti-rabbit HRP-labeled antibody (Abcam) was diluted in antibody dilution buffer. Finally, detection procedures were performed using ECL Advance TM western blotting detection kit and autoradiography was performed on Hyperfilm TM ECL (Amersham 175 Bioscience, Buckinghamshire, UK).

### Confocal microscopy

THP-1 macrophages were cultured in chamber slides in serum-free experimental medium or experimental medium with 40 ng/ml IL-6 or 50 ng/ml TNF-α or 25 µg/ml LDL in the absence or presence of glycosylation inhibitors (2.5 ng/ml kifunensine and 2.5 ng/ml swainsonine) for 24 h. After 24 h incubation, the cells were washed with PBS, fixed in 5% formalin solution for 30 min, permeablized with 0.25% of Triton X-100 for 15 min, and stained with rabbit polyclonal anti-human SCAP antibody produced by immunizing rabbits with the synthetic peptide PVDSDRKQGEPTEQC in our laboratory and mouse anti-Golgin antibody (Molecular Probes, Paisley, UK) for 1 h at room temperature. The cells were washed three times using PBS/Tween 20 over 15–30 min, finally visualization procedures were completed by dual-staining with goat anti-rabbit Fluor (green) 488 for SCAP and goat anti-mouse Fluor (red) 594 for Golgin (Molecular Probes) for 1 h at room temperature. Cells were examined with a confocal microscope (Olympus, Japan). The co-localization efficiency of SCAP with Golgi was quantified by Carl Zeiss Aim software.

### Protein Degradation

For protein stability assay, THP-1 macrophages were treated with 50 µmol/l Cycloheximide (CHX, Sigma, USA) in the presence or absence of cytokines for different time. Total proteins prepared for SCAP degradation were extracted as usual and an equal amount of protein was subjected to Western blotting.

### Statistical analysis

In all experiments, data were evaluated for significance by one-way ANOVA, followed by the multiple comparisons using LSD or Dunnett's T3 methods. Data were considered significant at P<0.05.

## Results

### The effects of inflammation and Golgi glycosylation enzyme inhibitors on intracellular cholesterol accumulation

**Figure 1:**
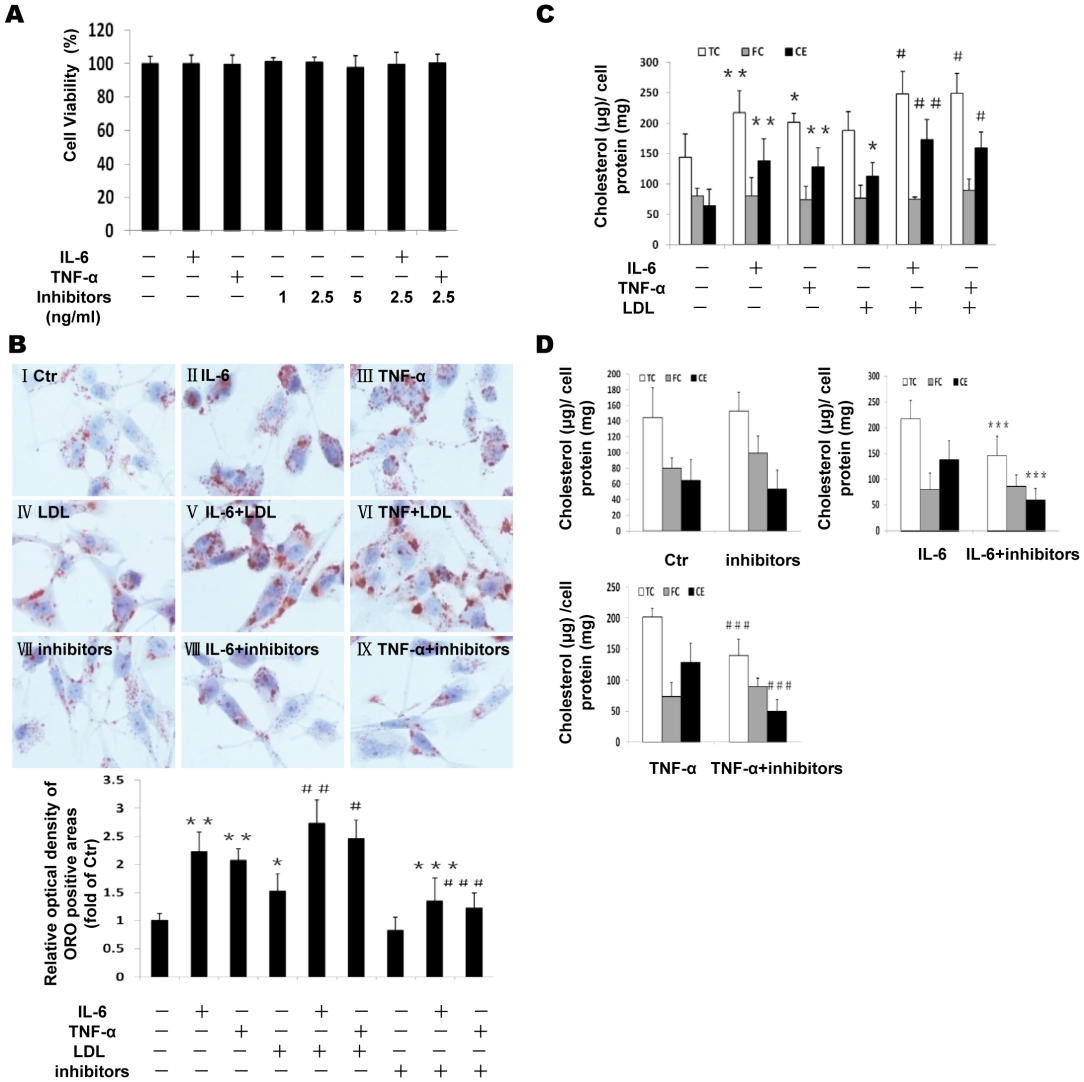
Effects of inflammation and Golgi glycosylation enzyme inhibitors on intracellular cholesterol accumulation in THP-1 macrophages. THP-1 macrophages were incubated in serum free medium for 24 h at 37°C. The medium was then replaced by fresh serum-free medium in the absence (control) (I) or presence of 40 ng/ml IL-6 (II) or 50 ng/ml TNF-α (III) or 25 µg/ml LDL (IV) or 40 ng/ml IL-6 plus 25 µg/ml LDL(V) or 50 ng/ml TNF-α plus 25 µg/ml LDL (VI) or glycosylation inhibitors (VII), or 40 ng/ml IL-6 plus inhibitors (VIII), or 50 ng/ml TNF-α plus inhibitors (IX) for 24 h at 37°C. Cell viability was determined using the MTT assay as described in Methods. Cell viability is expressed as the percentage of absorbance from treated cells compared to untreated cells. Graphs represent the average values (M±SD) of three independent experiments with triplicate biological measurements for each experiment (A). The cells were examined for lipid inclusions by Oil Red O (ORO) staining. The results are typical of those observed in four separate experiments (×400). Semi-quantitative analysis of ORO positive staining was performed by the Image-J software. Results represent mean± SD from 4 separate fields (B). Intracellular free cholesterol and total cholesterol were assayed as described in the Materials and Methods section. Values are means ± SD of duplicate wells from 4 experiments (C and D). ^*^P<0.05 vs control; ^**^P<0.01 vs control; ^***^P<0.01 vs IL-6; ^#^P<0.05 vs LDL; ^##^P<0.01 vs LDL; ^###^P<0.01 vs TNF-α.

Both cytokines and Golgi glycosylation inhibitors at the given concentrations used in the experiments did not change cell viability as demonstrated by MTT experiment (Figure 1A). We determined lipid accumulation in THP-1 macrophages in response to inflammatory cytokines and Golgi glycosylation enzyme inhibitors. Both inflammatory cytokines IL-6 and TNF-α increased Oil Red O staining in the absence (Figure 1B: II or III vs I) or presence of LDL (Figure 1B: V or VI vs IV) in THP-1 macrophages. Interestingly, effective glycosylation inhibitors (kifunensine and swainsonine) for α-mannosidase I and α-mannosidase II enzymes in Golgi reduced lipid accumulation induced by IL-6 and TNF-α in THP-1 macrophages (Figure 1B: VIII or IX vs VII). The histogram in Figure 1B shows the quantitative analysis of ORO positive staining. Intracellular cholesterol levels were also quantified using an enzymatic assay. TC was increased in cytokines treated cells in the absence or presence of LDL, which was attributed to the elevated CE but not FC (Figure 1C). The glycosylation inhibitors reduced intracellular CE and therefore TC induced by IL-6 and TNF-α (Figure 1D). These results suggest that intracellular cholesterol accumulation and foam cell formation induced by inflammation can be prevented by effective glycosylation inhibitors.

### The effects of inflammation on LDLr, HMGCoAR and SREBP2 expression

**Figure 2:**
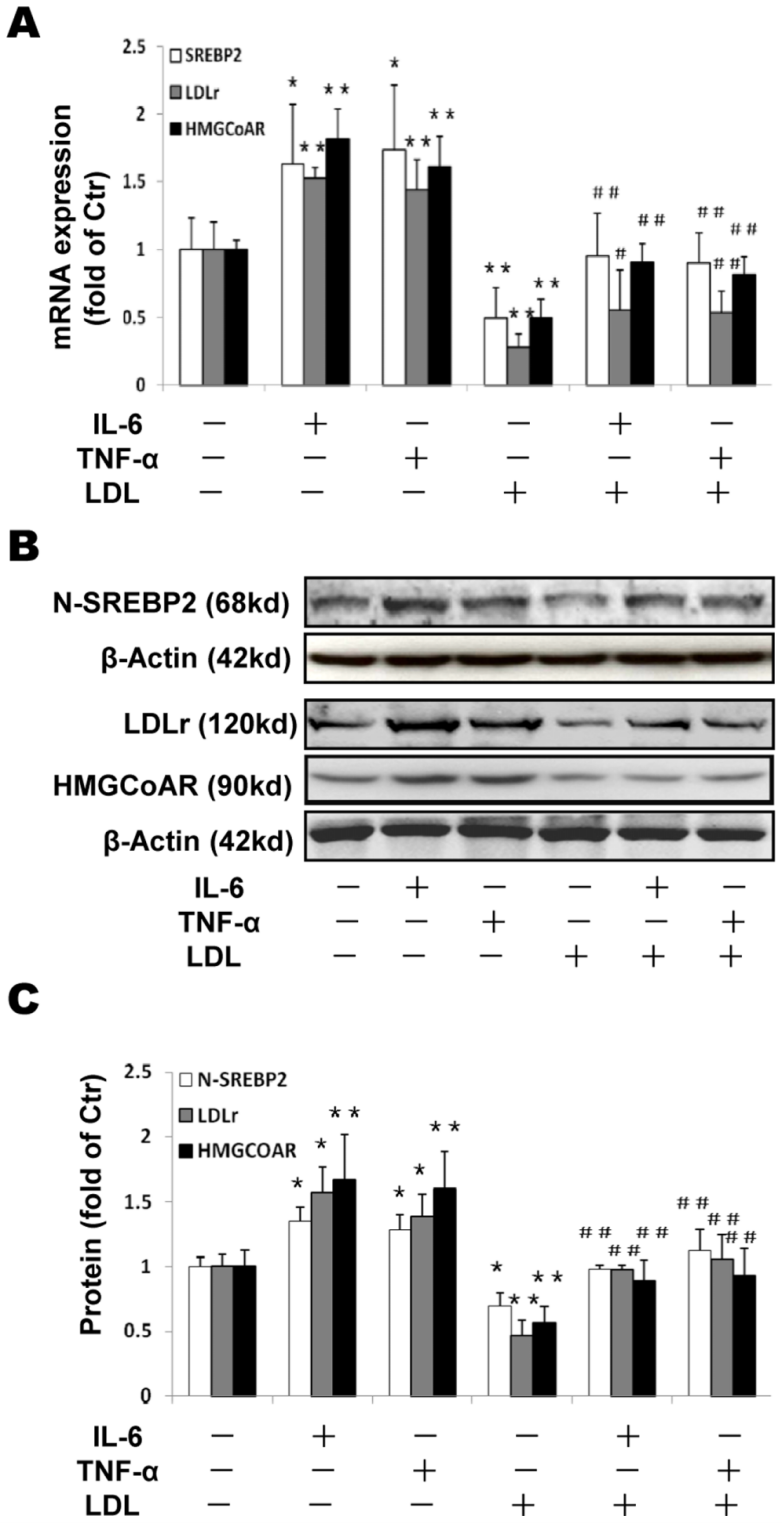
Effects of inflammation on LDLr, HMGCoAR and SREBP2 expression in THP-1 macrophages. THP-1 macrophages were incubated in serum free medium for 24 h at 37°C. The medium was then replaced by fresh serum-free medium in the absence (control) or presence of 40 ng/ml IL-6 or 50 ng/ml TNF-α or 25 µg/ml LDL alone or 40 ng/ml IL-6 plus 25 µg/ml LDL or 50 ng/ml TNF-α plus 25 µg/ml LDL for 24 h at 37°C. The mRNA levels were determined following the ΔΔ threshold cycle (Ct) protocol for real time RT-PCR as described in the Materials and Methods section. β-Actin served as a reference gene. Results represent the mean± SD from 4 experiments (A). The protein levels were examined by Western blotting (B). The histogram represents mean± SD of the densitometric scans of N-SREBP2, HMGCoAR and LDLr protein bands from four experiments, normalized by comparison with β-Actin and expressed as a percentage of control (C). ^*^P<0.05 vs control; ^**^P<0.01 vs control; ^#^P<0.05 vs LDL; ^##^ P<0.01 vs LDL.

We investigated effects of inflammatory cytokines on the expression of LDLr, HMGCoAR and SREBP2. Either IL-6 or TNF-α upregulated both mRNA (Figure 2A) and protein (Figure 2B and 2C) expression of LDLr and HMGCoAR. In addition, we checked protein levels of N-SREBP2 in nucleus (the active format of SREBP2 for LDLr and HMGCoAR transcription). Both cytokines increased N-SREBP2 levels (Figure 2B and 2C). LDL loading inhibited SREBP2, HMGCoAR and LDLr expression (Figure 2A, 2B and 2C) as expected (negative feedback regulation). However, cytokines overrode the suppression of these molecules by LDL (Figure 2A, 2B and 2C: LDL plus cytokines vs LDL alone). These results suggest that inflammation disrupts LDLr and HMGCoAR feedback regulation and increases LDL-cholesterol accumulation by activating SREBP pathway.

### The effects of Golgi glycosylation enzyme inhibitors on LDLr, HMGCoAR and SREBP2 expression

**Figure 3:**
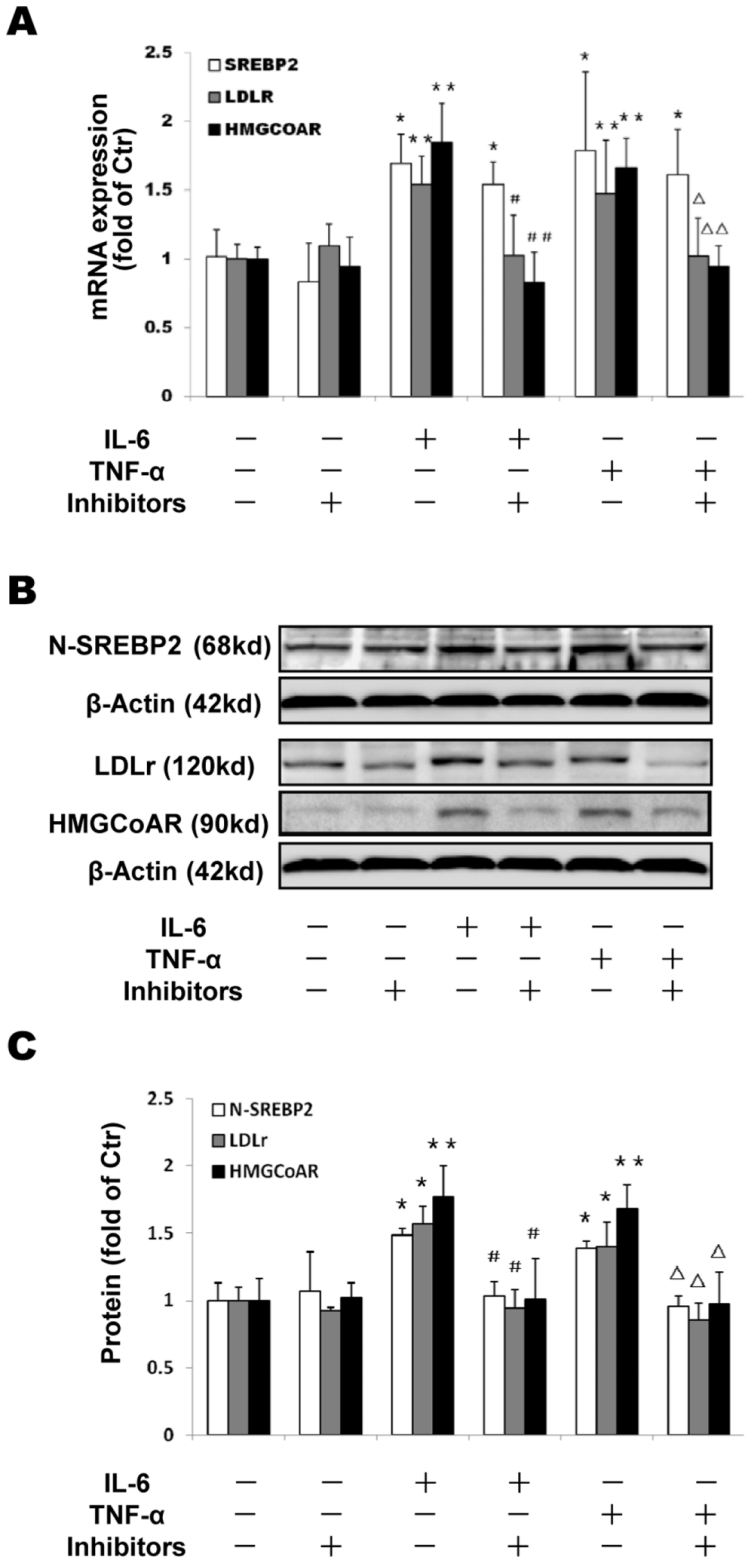
Effects of Golgi glycosylation enzyme inhibitors on LDLr, HMGCoAR and SREBP2 expression in THP-1 macrophages. THP-1 macrophages were incubated in serum free medium for 24 h at 37°C, and then exposed to glycosylation inhibitors (2.5 ng/ml kifunensine and 2.5 ng/ml swainsonine) in the absence or presence of 40 ng/ml IL-6 or 50 ng/ml TNF-α for 24 h. The cells were harvested and the mRNA expression of SREBP2 and LDLr were determined following the ΔΔ threshold cycle (Ct) protocol for real time RT-PCR as described in the Materials and Methods section. β-Actin served as the reference gene. Results represent the mean± SD from 4 experiments (A). The protein levels were examined by Western blotting (B). The histogram represents mean± SD of the densitometric scans of LDLr, HMGCoAR and N-SREBP2 protein bands from 4 experiments, normalized by comparison with β-Actin and expressed as a percentage of control (C). ^*^P<0.05 vs control; ^**^P<0.01 vs control; ^#^P<0.05 vs IL-6; ^##^P<0.01 vs IL-6; ^Δ^P<0.05 vs TNF-α; ^ΔΔ^P<0.01 vs TNF-α.

Interestingly, the Golgi glycosylation enzyme inhibitors decreased LDLr and HMGCoAR expression induced by the cytokines at both the mRNA and the protein levels (Figure 3A, 3B and 3C), in accordance with the reduction of the N-SREBP2 in the THP-1 macrophages (Figure 3B and 3C), suggesting that the Golgi enzyme inhibitors prevent inflammation- induced cholesterol accumulation.

### The effects of inflammation and Golgi glycosylation enzyme inhibitors on SCAP expression and intracellular translocation

**Figure 4:**
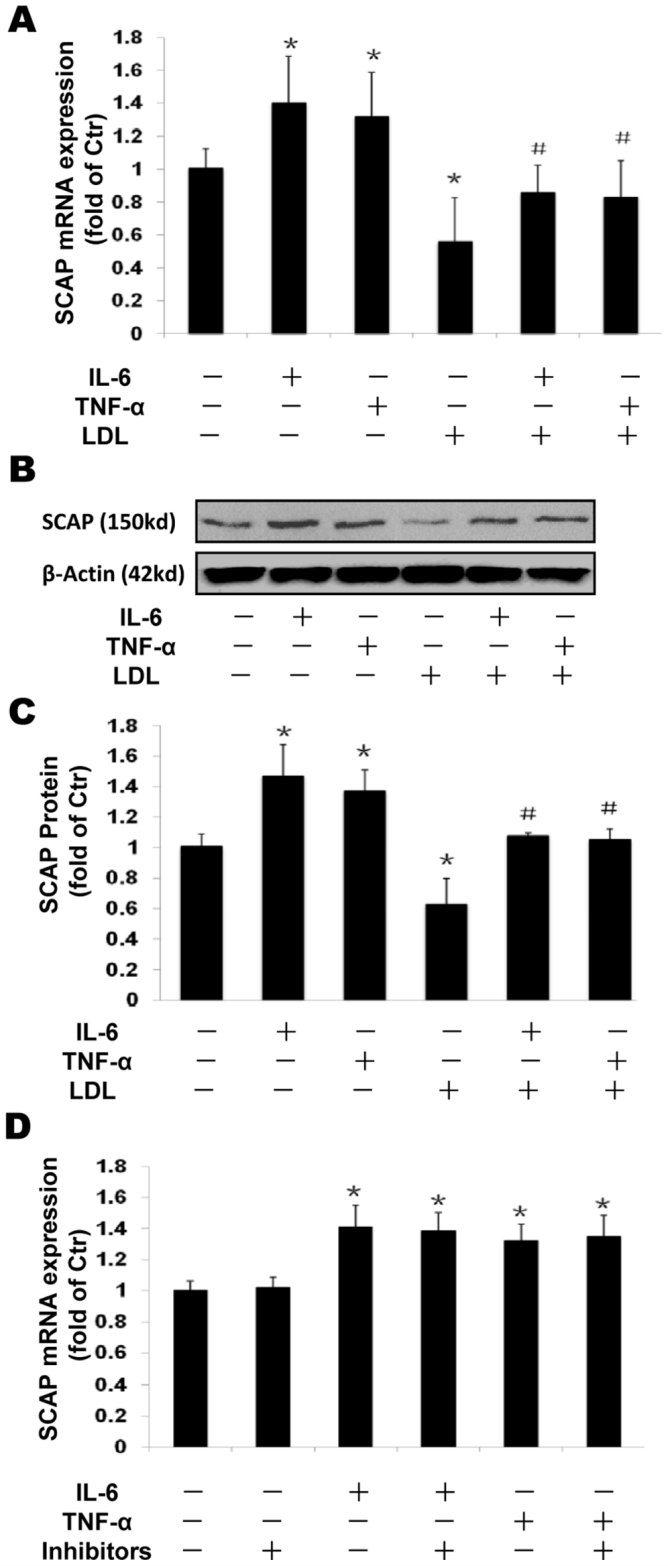
Effects of inflammation and Golgi glycosylation enzyme inhibitor on SCAP expression in THP-1 macrophages. THP-1 macrophages were incubated in serum free medium for 24 h at 37°C. The medium was then replaced by fresh serum-free medium in the absence (control) or presence of 40 ng/ml IL-6 or 50 ng/ml TNF-α or 25 µg/ml LDL alone or cytokine plus 25 µg/ml LDL or cytokine plus inhibitors for 24 h at 37°C. The mRNA levels were determined following the ΔΔ threshold cycle (Ct) protocol for real time RT-PCR as described in the Materials and Methods section. β-Actin served as a reference gene. Results represent the mean± SD from 4 experiments (A and D). The protein levels were examined by Western blotting (B). The histogram represents mean± SD of the densitometric scans of SCAP protein bands from four experiments, normalized by comparison with β-Actin and expressed as a percentage of control (C). ^*^P<0.05 vs control; ^#^P<0.05 vs LDL.

**Figure 5:**
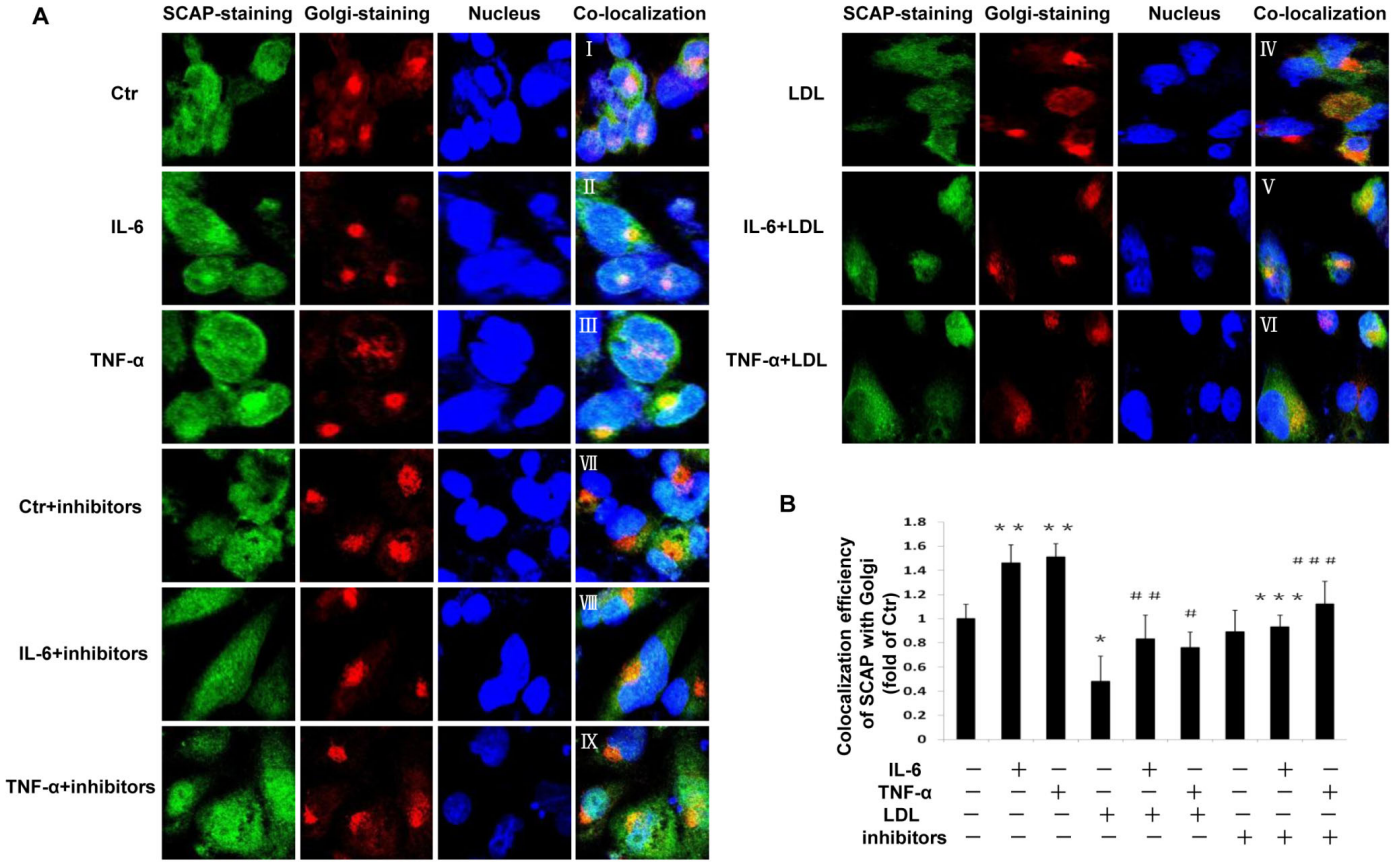
Effect of inflammatory cytokines and Golgi glycosylation enzyme inhibitors on protein translocation of SCAP from ER to Golgi in THP-1 macrophages. THP-1 macrophages plated in chamber slides were cultured in serum-free experimental medium or experimental medium with 40 ng/ml IL-6 or 50 ng/ml TNF-α in the absence or presence of 25 µg/ml LDL or glycosylation inhibitors (2.5 ng/ml kifunensine and 2.5 ng/ml swainsonine) for 24 h at 37°C. The translocation of SCAP from the ER to the Golgi was investigated using confocal microscopy after dual-staining as described in the Materials and Methods section (A). The co-localization efficiency of SCAP with Golgi was quantified by Carl Zeiss Aim software. Results represent mean±SD from 4 separate fields (B). ^*^P<0.05 vs control; ^**^P<0.01 vs control; ^***^P<0.01 vs IL-6; ^#^P<0.05 vs LDL; ^##^P<0.01 vs LDL; ^###^ P<0.05 vs TNF-α.

We investigated effects of inflammation on the expression of SCAP. Both IL-6 and TNF-α upregulated SCAP expression in mRNA (Figure 4A) and protein levels (Figure 4B and 4C), while native LDL loading significantly suppressed SCAP expression. However, the suppressive effect of native LDL was overridden by inflammatory cytokines. The Golgi glycosylation enzyme inhibitors showed no effect on SCAP mRNA expression (Figure 4D). Furthermore, we investigated SCAP translocation between the ER and the Golgi in THP-1 macrophages by confocal microscopy. Using dual immunofluorescence staining with anti-human antibodies of SCAP and Golgi, we demonstrated that both inflammatory cytokines IL-6 and TNF-α increased SCAP accumulation in the Golgi (Fig. 5 II &III vs I) even in the presence of native LDL loading which initially suppressed SCAP translocation from ER to Golgi (Fig. 5 V&VI vs IV). Interestingly, exposure to Golgi glycosylation inhibitors reduced IL-6 or TNF-α enhanced localization of SCAP in the Golgi (Fig. 5 VII vs I, VIII vs II and IX vs III).

### The effect of inflammation on SCAP stability

**Figure 6:**
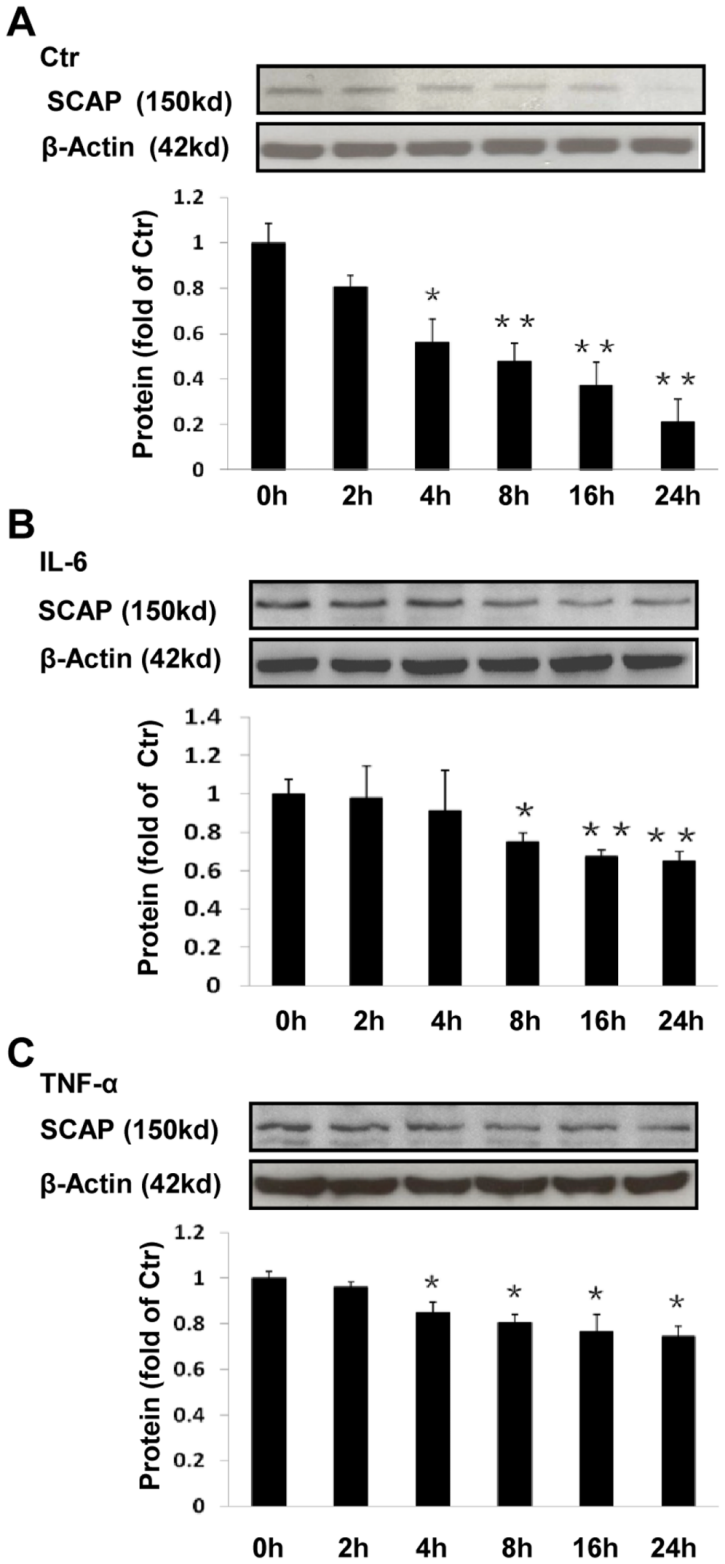
Effects of inflammation on SCAP stability in THP-1 macrophages. The cells were treated with or without 40/ml IL-6 or 50 ng/ml TNF-α for 24 h, and then chased in the presence of 50 µmol/l CHX for 0, 2, 4, 8, 16 and 24 h. The cells were lysed in equal volumes of buffer and protein levels plotted as a percentage of the SCAP remaining compared with the amount at 0 h. The histogram represents mean± SD of the densitometric scans of SCAP protein bands from 4 experiments, normalized to β- Actin and expressed as a percentage of control. ^*^P<0.05 vs 0 h; ^**^P<0.01 vs 0 h.

To evaluate the protein stability of SCAP in the absence or presence of inflammatory cytokine in THP-1 macrophages, we estimated the amount of protein remaining at various time points (0, 2, 4, 8, 16 or 24 h) after incubation with CHX, a protein synthesis inhibitor. We demonstrated that SCAP protein levels in THP-1 macrophages in serum free experimental medium declined in a time-dependent manner in the absence of inflammatory cytokine (Figure 6A). However, the decline of SCAP protein levels was prevented by either IL-6 or TNF-α at all time points (Figure 6B and 6C), suggesting that inflammation may increase SCAP protein stability.

### The effects of inflammation on Golgi enzymes expression

**Figure 7:**
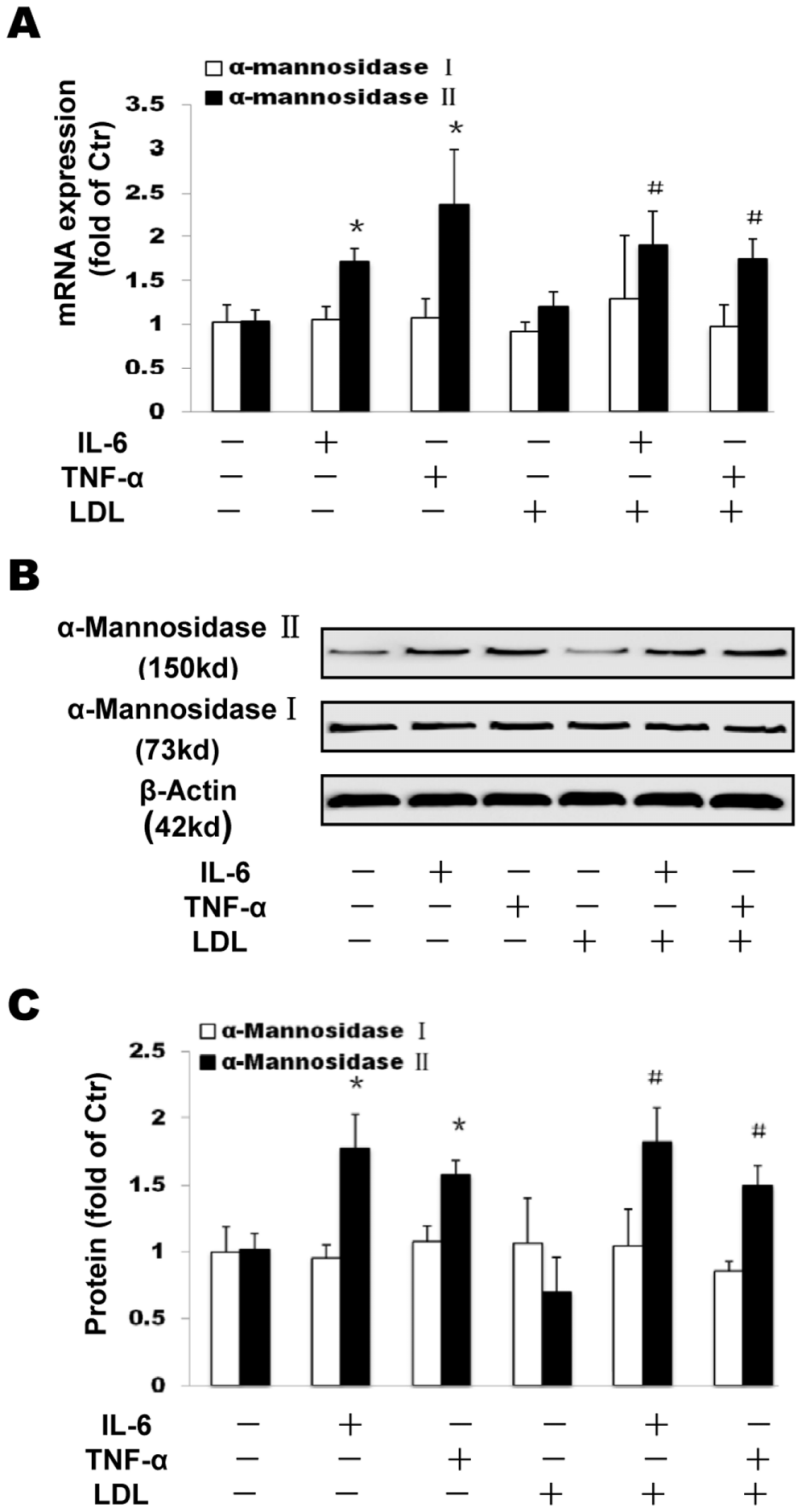
Effects of inflammation on Golgi enzymes α-mannosidase I and α-mannosidase II expression in THP-1 macrophages. THP-1 macrophages were incubated in serum free medium for 24 h at 37°C. The medium was then replaced by fresh serum-free medium in the absence (control) or presence of 40 ng/ml IL-6 or 50 ng/ml TNF-α or 25 µg/ml LDL alone or 40 ng/ml IL-6 plus 25 µg/ml LDL or 50 ng/ml TNF-α plus 25 µg/ml LDL for 24 h at 37°C. mRNA levels were determined following the ΔΔ threshold cycle (Ct) protocol for real time RT-PCR as described in the Materials and Methods section. β-Actin served as the reference gene. Results represent the mean± SD from 4 experiments (A). The protein levels were examined by Western blotting (B). The histogram represents mean± SD of the densitometric scans of proteins bands from four experiments, normalized by comparison with β-Actin and expressed as a percentage of control (C). ^*^ *P*<0.01 *vs* control; ^#^ *P*<0.05 *vs* LDL.

Here, we investigated effects of inflammation on the expression of the Golgi enzymes α-mannosidase I and α-mannosidase II, both of which are required for SCAP glycosylation. Both IL-6 and TNF-α significantly upregulated the expression of α-mannosidase II but not α-mannosidase I in the absence or presence of LDL at mRNA (Figure 7A) and protein levels (Figure 7B and 7C).

## Discussion

Both inflammation and dyslipidemia are aggressive factors in atherosclerosis [1], [3], [26]. Macrophages in the arterial wall, derived from the circulatory monocyte initially serve a protective function by removing cytotoxic and proinflammatory particles or apoptotic cells. However, they are highly heterogeneous cells that can rapidly change their functions in response to local microenvironmental signals, and progressive accumulation of macrophages and their uptake of lipoprotein-derived cholesterol [27]. In the present study, we used THP-1 derived macrophages activated by PMA, which are very similar to primary peripheral blood mononuclear cells in response to inflammatory cytokines to study the role of inflammation on LDL metabolism. Our data demonstrated that inflammatory cytokine either IL-6 or TNF-α, which are both biomarkers of chronic systemic inflammation, promoted native LDL accumulation in THP-1 macrophages. Since all experimental media in this study contained the antioxidants EDTA and BHT, both of which powerfully prevent oxidation of LDL [7], [8]. In this case, it is highly unlikely that a suitable ligand for the scavenger receptor was present in current experimental conditions. In addition to the results from ORO staining in this study, the intracellular cholesterol quantitative measurements by enzymatic methods also showed that inflammatory cytokines increased TC and CE contents in THP-1 macrophages, though SREBP1-mediated fatty acids synthesis might also be increased. Interestingly, CE, but not FC, was elevated dramatically by administration of inflammatory cytokines. This result may be attributed to the elevated activity and expression of Acyl-coenzyme A: cholesterol acyltransferase 1 (ACAT1), which is the key intracellular enzyme catalyzing the formation of cholesteryl esters, under the influence of inflammatory cytokine and LDL [7]. The development of macrophage-derived foam cells that contain massive amounts of cholesteryl esters becomes a hallmark of early stage of atherosclerotic lesions [28], [29].

In this study, we demonstrated that inflammatory cytokines IL-6 and TNF-α increased both mRNA and protein expression of SCAP. It seems that cholesterol level may be decreased in the ER under inflammatory stress since the elevated ACAT activity converts more FC to CE, This may result in insufficient cholesterol content to retain SCAP in the ER in the presence of inflammation [30], [31], [7]. Therefore, increased SCAP expression results in SCAP translocation from the ER to the Golgi as demonstrated in Figure 5. This abnormal escape of the SCAP shuttles more SREBP2 to the Golgi for cleavages, which produces extra N-SREBP2, leading to up-regulation of LDLr and HMGCoAR even in the presence of a high concentration of LDL which normally inhibits this process.

Glycosylation is a frequent post-translational modification of proteins which modulates a variety of biological functions [22], [32]. Two Golgi enzymes (α-mannosidase I and α-mannosidase II) are considered responsible for SCAP glycosylation [15]. The α-mannosidase II is an enzyme in the N-linked glycosylation pathway that is responsible for the removal of the terminal α1–3-and α1–6-linked mannose residues prior to the addition of N-acetylglucosamine (GlcNAc) residues which are required for the synthesis of complex-type asparagine (N)-linked glycans [33]. In this study, we demonstrated that in inflammatory cytokine treated macrophages, the mRNA and protein expression of α-mannosidase II (not α-mannosidase I) was increased, accompanying with increased lipid droplet deposition and activation of SREBP2/LDLr pathway. Meanwhile, the increased SCAP stability by cytokines was resulted from the increased SCAP Golgi glycosylation by α-mannosidase II. Interestingly, Golgi glycosylation enzyme inhibitors strikingly attenuated the translocation of SCAP from the ER to the Golgi and reduced the levels of LDLr, HMGCoAR and N-SREBP2, preventing lipid droplet deposition induced by cytokines. It seems that glycosylation of SCAP may prevent SCAP degradation and prolong SCAP half-life, facilitating SCAP recycling between the ER and the Golgi, and activating SREBP2/LDLr pathway. Interestingly, it showed no significant effects of inhibitors on SREBP2, LDLR and HMGCoAR mRNA level in the non-inflamed group. This might suggest that the effect of inhibitors on the downstream molecules are mild in physical conditions, however, the effect can be strikingly amplified in inflamed conditions. The mechanisms by which inflammation regulates SCAP gene expression and posttranslational modification are not clear. We have previously demonstrated that knocking down of MyD88 or using IKK inhibitor in THP-1 derived macrophages attenuates the increase of SCAP and its down-stream molecules (N-SREBP2 and LDLr) by inflammation, suggesting a crosstalk between inflammation and SCAP expression pathway [34].

Taken together, we demonstrated that inflammation increases expression and potential glycosylation of SCAP in the Golgi by increasing expression of Golgi α-mannosidase II, causing an abnormal translocation of SCAP from the ER to the Golgi and enhancing recycling of SCAP complex between the ER and the Golgi. These processes increase SREBP2 cleavages and produce more N-SREBP2 which consequently activates LDLr and HMGCoAR expression and lipid accumulation in THP-1 macrophages. These results may improve our understanding of the molecular mechanisms of atherosclerosis and also suggest that anti-inflammation or Golgi glycosylation enzyme inhibitors may be useful adjunctive therapeutic agents in the management of atherosclerosis.
